# Visualizing the vascular-cellular microenvironment in lung cancer brain metastasis via multiparametric fusion of DCE-MRI and DWI

**DOI:** 10.3389/fonc.2025.1645589

**Published:** 2025-10-21

**Authors:** Yonglong Li, Zhen Li, Haotian Wang, Zhigang Pei, Xiufu Zhang, Jun Zhou, Chunrong Wu, Ruipeng Liang

**Affiliations:** 1 Department of Radiology, Chongqing University Jiangjin Hospital, Jiangjin, Chongqing, China; 2 Department of Emergency Medicine, Chongqing University Jiangjin Hospital, Jiangjin, Chongqing, China; 3 Department of Pathology, Chongqing University Jiangjin Hospital, Jiangjin, Chongqing, China; 4 Department of Oncology, Chongqing University Jiangjin Hospital, Jiangjin, Chongqing, China

**Keywords:** diffusion-weighted imaging (DWI), dynamic-contrast-enhanced magnetic resonance imaging, lung cancer, brain metastasis (BM), microenvironment

## Abstract

**Background:**

The heterogeneity of the lung cancer brain metastasis (LCBM) microenvironment limits therapeutic efficacy, while invasive pathological biopsies fail to dynamically assess brain metastasis (BM) comprehensively. Non-invasive imaging techniques thus hold clinical value for visualizing the LCBM microenvironment. This study aimed to achieve non-invasive quantitative analysis of vascular function and cellular structures in LCBM using multiparametric Dynamic Contrast-Enhanced Magnetic Resonance Imaging (DCE-MRI) and Diffusion-Weighted Imaging (DWI).

**Methods:**

A prospective cohort of 114 LCBM patients (63 lung adenocarcinoma [LUAD]-BM, 28 lung squamous cell carcinoma [LUSC]-BM, 23 small cell lung cancer [SCLC]-BM) underwent DCE-MRI and DWI on a 3.0T MRI scanner. Parameters including volume transfer constant (Ktrans), rate constant (Kep), extravascular extracellular volume (Ve), and plasma volume fraction (Vp) were derived using the Extended Tofts model. Group differences were analyzed via Mann-Whitney U test, diagnostic efficacy via ROC curves, and parameter interactions via multivariate logistic and linear regression.

**Results:**

ADC distinguished SCLC-BM from NSCLC-BM with AUC=0.891 (specificity=95.05% at 752.4×10^−6^mm²/s), while Ktrans differentiated LUAD-BM from LUSC-BM with AUC=0.998 (sensitivity=98.48%, specificity=97.79% at 157 min^−1^/1000). Microenvironmental profiles: LUAD-BM showed high Vp (51.50/1000) and Ktrans (424.8 min^−1^/1000); LUSC-BM had low Ktrans (61.15 min^−1^/1000) and medium ADC (1163×10^−6^mm²/s); SCLC-BM exhibited high cellular density (ADC=661×10^−6^mm²/s) and abnormal contrast kinetics (high Kep, low Vp). Vp and ADC were identified as independent predictors for LUAD-BM and SCLC-BM, respectively. Parameter interactions varied by subtype: ADC in LUAD-BM correlated with Kep and Delay Time; in LUSC-BM, with Ktrans and Ve; and in SCLC-BM, showed weaker vascular associations. Ktrans regulation involved distinct parameter contributions across subtypes.

**Conclusion:**

A DCE-MRI-DWI “Vascular-Cellular Microenvironment Visualization Model” was established, revealing distinct profiles: high microvascular density/permeability in LUAD-BM, low permeability/medium cellularity in LUSC-BM, and high cellularity/abnormal contrast kinetics in SCLC-BM. This validates multimodal imaging for characterizing LCBM heterogeneity and provides insights into tumor angiogenesis, cellular density, and BBB regulation, supporting microenvironment-targeted therapy.

## Introduction

Lung Cancer Brain Metastasis (LCBM) is a central clinical challenge leading to shortened patient survival and diminished quality of life (1, 2). Although targeted therapy and immunotherapy have significantly prolonged the overall survival of lung cancer patients (3), the dynamic evolution of the Brain Metastasis (BM) microenvironment—including disruption of the blood-brain barrier (BBB) (4), tumor vascularization patterns (5), and heterogeneity in extracellular matrix remodeling (6)—remains a critical factor limiting therapeutic efficacy. Traditional pathological biopsies face challenges in dynamically assessing the comprehensive microenvironment of BM due to the high risks associated with cranial surgery, significant sampling bias (which captures only local lesion characteristics), and the time lag in postoperative pathological analysis. Consequently, the development of noninvasive, reproducible imaging biomarkers to holistically characterize the vascular-cellular microenvironment of LCBM has become a pivotal research direction in the era of precision medicine.

The microenvironment heterogeneity of LCBM serves as the core biological basis for its aggressiveness and therapy resistance. This heterogeneity encompasses multidimensional interactions, including vascularization patterns (e.g., microvascular density and permeability), cellular structural characteristics (e.g., cellular density and extracellular space), and the functional status of the BBB/Blood-Tumor Barrier (BTB) (e.g., tight junction protein expression and efflux pump activity). Traditional pathological biopsy, due to its invasiveness and sampling limitations, fails to meet the dynamic assessment requirements for microenvironment heterogeneity in BM.In recent years, Magnetic Resonance Imaging (MRI) has emerged as a crucial tool in LCBM diagnosis and treatment owing to its multimodal functional imaging capabilities (7–10). Among these techniques, Diffusion-Weighted Imaging (DWI) quantifies the restricted diffusion of water molecules within tissues via the Apparent Diffusion Coefficient (ADC), offering an intuitive reflection of tumor cellular density and the physical properties of the extracellular matrix (11, 12). Conversely, Dynamic Contrast-Enhanced Magnetic Resonance Imaging (DCE-MRI) noninvasively characterizes multidimensional micro-pathophysiological information—such as tumor perfusion, vascular permeability, and microvascular content—by quantifying parameters including the Volume Transfer Constant (K^trans^), Rate Constant (K_ep_), Extravascular Extracellular Volume (Ve), and Plasma Volume (Vp) (3, 13, 14).Theoretically, these two modalities possess complementary analytical potential, providing tumor microenvironment insights from the dual dimensions of “cellular structure” and “vascular function.” However, current research utilizing imaging techniques to evaluate LCBM microenvironment heterogeneity faces significant limitations. One major drawback is that conventional imaging analysis relies on single-parameter thresholds, which inadequately capture the heterogeneous distribution of the tumor microenvironment (15, 16). Another issue is that most DCE-MRI studies focus on primary lung cancer (12), and conclusions drawn from body-based analyses cannot be directly extrapolated to the brain due to its unique physiological structure (e.g., blood supply patterns) (8).

To address the aforementioned issues, this study proposes a multiparameter fusion method based on the combination of DCE-MRI and DWI. By quantifying parameters such as Ktrans, Vp, and ADC, we constructed a visual model of the vascular-cellular microenvironment in LCBM. Specifically, this research aims to elucidate the characteristic profiles of DCE-MRI/DWI parameters across different histopathological subtypes of LCBM and reveal the features of the vascular-cellular microenvironment in LCBM. The innovation of this study lies in the first integration of vascular functional parameters from DCE-MRI with structural parameters from DWI to establish a visual model of the vascular-cellular microenvironment in LCBM. This model provides an imaging basis for the precise subtyping of LCBM and the selection of anti-angiogenic therapy targets, thereby offering a novel tool for the pathophysiological assessment of LCBM and laying an imaging foundation for individualized treatment strategies targeting LCBM pathophysiology.

## Methods

### Patients

This study was approved by the Hospital Ethics Review Committee (Approval No. KY2023073). All participants were fully informed of the research objectives and provided written informed consent. The research process adhered to the ethical principles outlined in the Declaration of Helsinki. A total of 114 patients with LCBM who underwent MRI examinations were prospectively enrolled from August 2022 to June 2025. Based on the pathological type of the primary lung cancer, the patients were divided into three groups: the lung adenocarcinoma (LUAD) group (n=63, 93 brain metastases [BM]), the lung squamous cell carcinoma (LUSC) group (n=28, 44 BM), and the small cell lung cancer (SCLC) group (n=23, 38 BM).Inclusion Criteria:① Primary pathological type of lung cancer confirmed by needle biopsy or surgical pathology;② Complete clinical and MRI data.Exclusion Criteria:① Undergoing radiotherapy, chemotherapy, or anti-angiogenic therapy;② BM located in the meninges, brainstem, or cerebellum;③ Maximum diameter of BM less than 0.5 cm;④ History of cranial surgery;⑤ Concurrent central nervous system diseases.

### Examination method

The examinations were performed using a United Imaging 3.0T MRI scanner (UMR780).Conventional sequence scanning, including DWI, was conducted first, followed by DCE-MRI sequence scanning. The DCE-MRI protocol comprised a multi flip-angle scanning sequence with five flip angles and subsequent multi-phase dynamic enhancement sequences.The multi flip-angle scanning sequence included five single-phase flip-angle scans with flip angles of 3°, 6°, 9°, 12°, and 15°, respectively. The matrix size was 112 × 100, the field of view (FOV) was 230 mm × 200 mm, the repetition time (TR)/echo time (TE) was 4.11 ms/1.84 ms, and the number of excitations (NEX) was 1. A total of 20 slices were acquired, with a slice thickness of 6 mm and a slice gap of 0 mm. The slice direction offset was set to 2.For the multi-phase dynamic enhancement sequence, the flip angle was set to 10°, TR/TE to 2.51 ms/0.92 ms, and the temporal resolution to 3 s. Other parameters, including the number of slices, slice thickness, slice gap, slice direction offset, matrix size, and FOV, were consistent with those of the single-phase flip-angle scanning sequence. A total of 90 phases were scanned, yielding 3,600 images. The total scan time for dynamic perfusion was 253 s. The contrast agent (Gadobutrol) was administered intravenously via the antecubital vein using a high-pressure injector at a rate of 3 mL/s at the end of phase 5 and the beginning of phase 6 of the dynamic enhancement sequence. Immediately after contrast agent injection, 20 mL of 0.9% saline solution was infused at the same rate.

### Image analysis

This study employed the Extended Tofts two-compartment model for post-processing DCE-MRI data. First, motion correction was performed to eliminate respiratory motion artifacts. The middle cerebral artery was selected as the arterial input function, and the superior sagittal sinus as the venous output function to derive time-signal intensity curves. On the maximum cross-section of lesions in enhanced T1-weighted imaging (T1WI) sequences, regions of interest (ROIs) were manually delineated. For ring-enhancing or large-volume lesions, a multi-region ROI delineation strategy (3–5 ROIs per lesion) was adopted to adequately reflect the heterogeneity of brain metastases (BM), strictly avoiding necrotic, cystic, and hemorrhagic areas.

Two radiologists independently delineated tumor ROIs (excluding necrotic, cystic, and hemorrhagic regions). Inter-observer agreement was evaluated using a two-way mixed-effects model. After validation, the intraclass correlation coefficient (ICC) for all parameters was ≥0.8. Data from the senior radiologist were ultimately used for analysis (ROI counts: 136 for the LUAD group, 66 for the LUSC group, and 62 for the SCLC group).

### Statistical analysis

This study employed SPSS 22.0 for data processing and regression analysis, and GraphPad Prism 9 for graph generation and receiver operating characteristic (ROC) curve analysis. Quantitative data were first subjected to normality testing using the Kolmogorov-Smirnov test. Normally distributed data were expressed as mean ± standard deviation (SD), and pairwise comparisons were performed using the LSD-t test. Non-normally distributed data were presented as median (interquartile range) [M (P25, P75)], and pairwise comparisons were conducted using the Mann-Whitney U test.The diagnostic efficacy of each parameter was evaluated using ROC curves, with the area under the curve (AUC), optimal Youden index, sensitivity, and specificity calculated (AUC ≥ 0.7 indicating good performance; AUC ≥ 0.8 indicating excellent performance). A multivariate binary logistic regression model was constructed with pathological type as the dependent variable and ADC values and DCE-MRI parameters as independent variables. After adjusting for confounding factors, the independent predictive value of each parameter for histopathological subtypes was assessed (results expressed as β, odds ratio [OR], and 95% confidence interval [CI]). Additionally, multiple linear regression models were built with ADC values and K^trans^ as dependent variables to analyze parameter interaction mechanisms. All statistical tests were two-sided, and a p-value < 0.05 was considered statistically significant.

## Results

### Differences in ADC values and DCE-MRI parameters among different pathological types of LCBM

**Figure 1:**
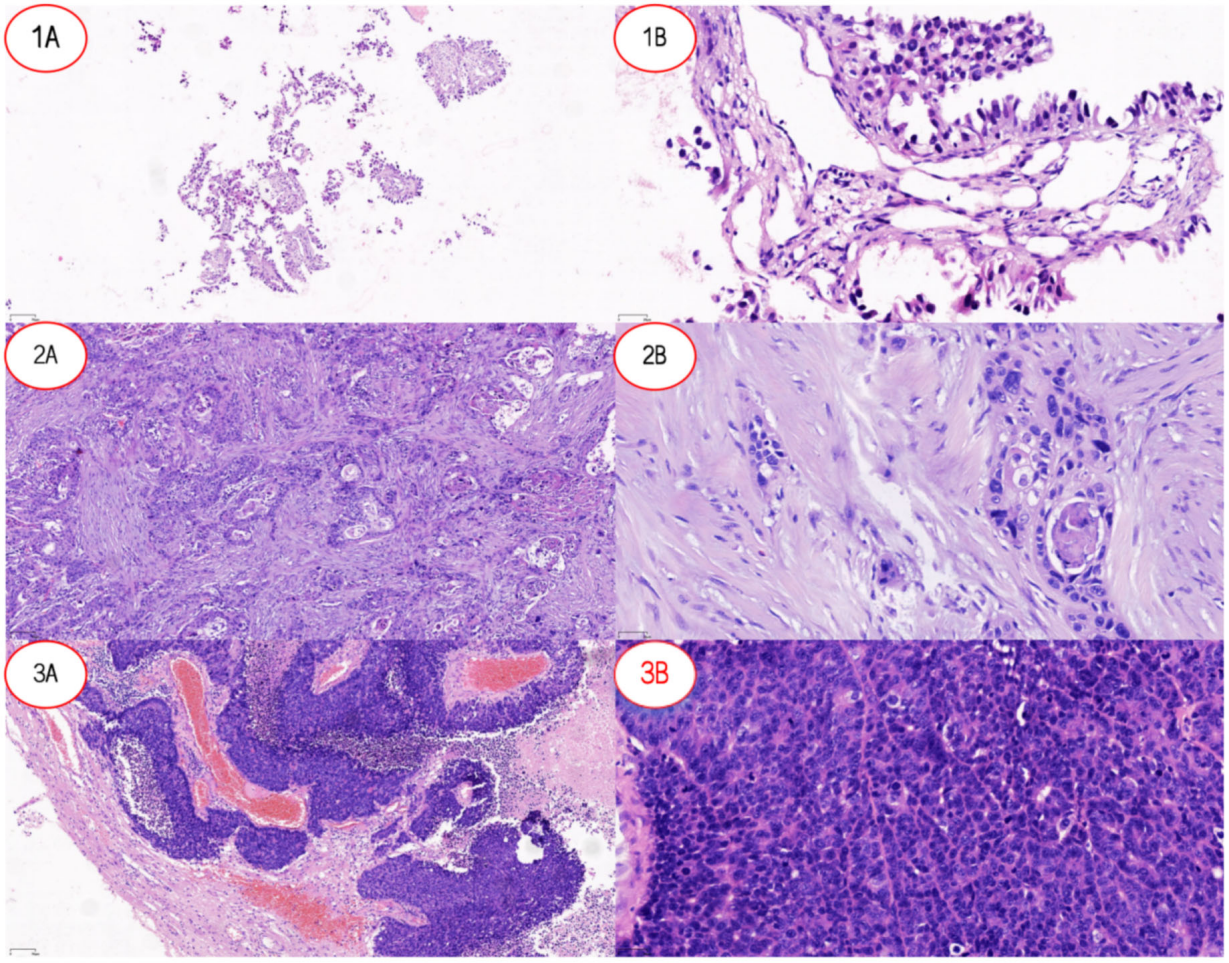
Pathological features of lung cancer and lung cancer brain metastases. **(1A)** Low-power view (×10) of LUAD-BM showing tumor tissue arranged in an acinar pattern with loose distribution. **(1B)** High-power view (×20) of LUAD-BM demonstrating columnar tumor cells forming glandular structures, consistent with adenocarcinoma differentiation. **(2A)** Low-power view (×10) of LUSC revealing nested tumor growth with well-defined stromal boundaries. **(2B)** High-power view (×20) of LUSC highlighting polygonal tumor cells with focal keratinization, characteristic of squamous cell carcinoma morphology. **(3A)** Low-power view (×10) of SCLC-BM displaying densely infiltrating tumor cells with stromal hemorrhage. **(3B)** High-power view (×20) of SCLC-BM showing small tumor cells with hyperchromatic nuclei (high nuclear-to-cytoplasmic ratio) diffusely distributed, typical of small cell carcinoma. A*bbreviations:* LUAD-BM, lung adenocarcinoma brain metastasis; LUSC, lung squamous cell carcinoma; SCLC-BM, small cell lung cancer brain metastasis.

Significant heterogeneity in ADC values and DCE-MRI parameters was observed across three pathological types of LCBM, as detailed in **Table 1**, with pathophysiological differences corroborated by pathological examination (**Figure 1**). The ADC value for lung adenocarcinoma brain metastases (LUAD-BM) was 1168 (838–1358) ×10^-6^ mm²/s, while for lung squamous cell carcinoma brain metastases (LUSC-BM) it was 1163 (885.4–1484) ×10^-6^ mm²/s, showing no significant difference between them (p=0.431). However, both values were significantly higher than that of small cell lung cancer brain metastases (SCLC-BM) at 661 (509.4–807.2) ×10^-6^ mm²/s (p<0.0001), indicating higher cellular density in SCLC-BM. Among DCE-MRI parameters, K^trans^ was highest in LUAD-BM [424.8 (271.5–525.2) min^-^¹/1000], significantly exceeding SCLC-BM [124.7 (64.58–154.4) min^-^¹/1000] and LUSC-BM [61.15 (44.98–102) min^-^¹/1000] (all p<0.0001). K_ep_ followed a similar trend: LUAD-BM [1250 (843–1695) min^-^¹/1000] > SCLC-BM [671.1 (520.8–861.3) min^-^¹/1000] > LUSC-BM [456.3 (368.1–571.3) min^-^¹/1000] (all p<0.0001). Ve in LUAD-BM [294.3 (215.7–478.7)/1000] was significantly greater than in both LUSC-BM [144.1 (96.63–215.5)/1000] and SCLC-BM [160.9 (90.2–228.6)/1000] (all p<0.0001), though no difference existed between LUSC-BM and SCLC-BM (p=0.71). Vp decreased progressively: LUAD-BM [51.50 (42.1–70.5)/1000] > SCLC-BM [35.45 (25.05–46.93)/1000] > LUSC-BM [15.55 (12.18–24.35)/1000] (all p<0.0001). Enhancement-related parameters—including initial area under the curve at 90 seconds (iAUC_90_), contrast enhancement ratio (CER), and maximum slope of enhancement (Max. Slope)—were significantly elevated in LUAD-BM compared to both other groups (all p<0.0001), while no significant differences emerged between LUSC-BM and SCLC-BM for these metrics. Time to peak enhancement (Delay) also varied significantly (all p<0.05), with LUSC-BM [5.9 (4.75–6.8) s] > LUAD-BM [4.9 (4.2–6.1) s] > SCLC-BM [4.45 (3.5–5.4) s].

**Table 1:** Compares ADC values and DCE-MRI parameters across distinct pathological subtypes of LCBM.

Parameters	LUAD-BM(n=136)	LUSC-BM(n=66)	SCLC-BM(n=62)	U	*P*
ADC(10^-6^mm²/s)	**1168(838, 1358)**	**1163(885.4, 1484)**	**661(509.4, 807.2)**	**4180^$^,871^&^,** **495^¥^**	**0.431^$^,<0.0001^&^,** **<0.0001^¥^**
K^trans^(min^-1^/1000)	**424.8(271.5, 525.2)**	**61.15(44.98, 102)**	**124.7(64.58, 154.4)**	**19^$^,213^&^,** **1087^¥^**	**<0.0001^$^,<0.0001^&^,<0.0001^¥^**
K_ep_(min^-1^/1000)	**1250(843, 1695)**	**456.3(368.1, 571.3)**	**671.1(520.8, 861.3)**	**345^$^,1402^&^,** **882.5^¥^**	**<0.0001^$^,<0.0001^&^,<0.0001^¥^**
Ve(1/1000)	**294.3(215.7, 478.7)**	**144.1(96.63, 215.5)**	**160.9(90.2, 228.6)**	**1366^$^,1640^&^,1968^¥^**	**<0.0001^$^,<0.0001^&^,0.71^¥^**
Vp(1/1000)	**51.50(42.1, 70.5)**	**15.55(12.18, 24.35)**	**35.45(25.05, 46.93)**	**217^$^,1677^&^,** **653.5^¥^**	**<0.0001^$^,<0.0001^&^,<0.0001^¥^**
iAUC90(min·mmol/L/100)	**59.85(50.33, 74.7)**	**17.60(11.08, 29.13)**	**21.85(13.1, 26.25)**	**553^$^,119.5^&^,1856^¥^**	**<0.0001^$^,<0.0001^&^,0.367^¥^**
CER(1/100)	**240.2(214.5, 290.1)**	**106(58.03, 164.4)**	**111.5(73.25, 154.1)**	**1032^$^,482.5^&^,2015^¥^**	**<0.0001^$^,<0.0001^&^,0.884^¥^**
Max.Slope(mmol·min^-^¹/100)	**213.6(172.9, 252.3)**	**55.65(33.83, 90.55)**	**70(53.1, 82.73)**	**424^$^,22.5^&^,** **1775^¥^**	**<0.0001^$^,<0.0001^&^,0.794^¥^**
Delay(s)	**4.9(4.2, 6.1)**	**5.9(4.75, 6.8)**	**4.45(3.5, 5.4)**	**3379^$^,3007^&^,1035^¥^**	**0.004^$^,0.001^&^,<0.0001^¥^**

Statistical annotations denote pairwise comparisons: “$” indicates LUAD-BM versus LUSC-BM; “&” denotes LUAD-BM versus SCLC-BM; “¥” represents LUSC-BM versus SCLC-BM. All tabulated data followed non-normal distributions and are presented as medians with interquartile ranges [M (P25, P75)].DCE-MRI, Dynamic Contrast-Enhanced Magnetic Resonance Imaging; ADC, Apparent Diffusion Coefficient; LUAD-BM, Lung Adenocarcinoma Brain Metastasis; LUSC-BM, Lung Squamous Cell Carcinoma Brain Metastasis; SCLC-BM, Small Cell Lung Cancer Brain Metastasis; K^trans^, Volume Transfer Constant; K_ep_, Rate Constant; Ve, Vascular Extracellular Volume Fraction; Vp, Plasma Volume Fraction; iAUC_90_, Initial area under the curve at 90 seconds; CER, Contrast Enhancement Ratio; Max.Slope, Maximum Slope; Delay, Delay Time. Bold values indicate statistically significant results.

### Diagnostic value of DCE-MRI and DWI parameters in differentiating pathological subtypes of LCBM

**Figure 2:**
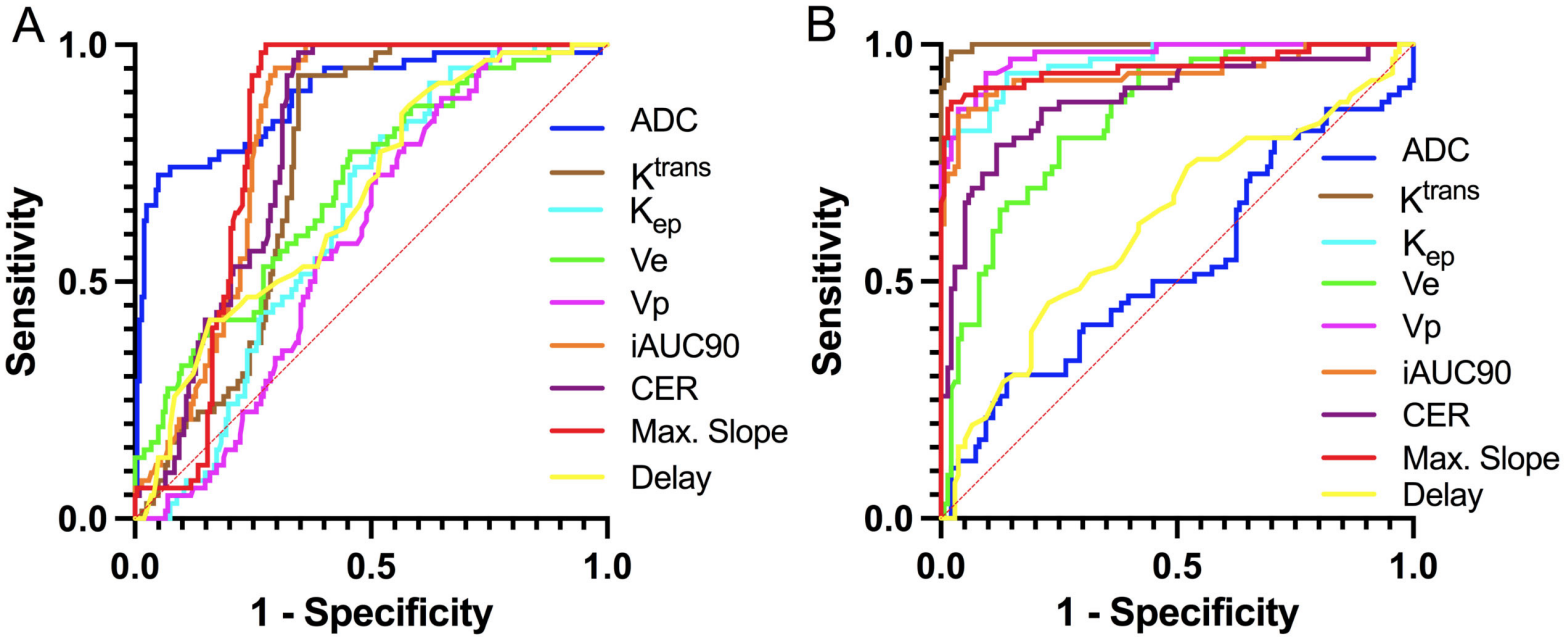
ROC curves for discriminating histopathological subtypes of LCBM based on DCE-MRI and DWI parameters. **(A)** SCLC vs. NSCLC; **(B)** CLC-BM vs. NSCLC-BM.

For two critical pathological differentiation tasks in LCBM—distinguishing SCLC from non-small cell lung cancer (NSCLC), and LUAD from LUSC—this study systematically evaluated the diagnostic efficacy of DCE-MRI parameters and ADC values. The results, summarized in **Tables 2** and **3** and illustrated in **Figure 2**, are as follows:

**Table 2:** Histopathological differential diagnosis of SCLC-BM and NSCLC-BM.

Parameters	AUC	95% CI	*P*	Cutoff value	Sensitivity	Specificity
ADC(10^-6^mm²/s)	**0.891**	**0.841-0.941**	**<0.0001**	**752.4**	**72.58%**	**95.05%**
K^trans^(min^-1^/1000)	**0.743**	**0.686-0.801**	**<0.0001**	**176.1**	**93.55%**	**64.36%**
K_ep_(min^-1^/1000)	**0.632**	**0.564-0.670**	**0.0017**	**1202**	**91.94%**	**37.13%**
Ve(1/1000)	**0.699**	**0.628-0.771**	**<0.0001**	**232.5**	**77.42%**	**53.47%**
Vp(1/1000)	**0.592**	**0.522-0.661**	**0.0293**	**51.40**	**88.71%**	**34.16%**
iAUC90(min·mmol/L/100)	**0.812**	**0.762-0.862**	**<0.0001**	**35.90**	**95.16%**	**70.30%**
CER(1/100)	**0.796**	**0.744-0.847**	**<0.0001**	**171.7**	**98.39%**	**65.35%**
Max.Slope (mmol·min^-^¹/100)	**0.813**	**0.763-0.864**	**<0.0001**	**108.8**	**100.00%**	**72.28%**
Delay(s)	**0.677**	**0.606-0.749**	**<0.0001**	**5.75**	**87.10%**	**42.08%**

SCLC-BM, Small Cell Lung Cancer Brain Metastasis; NSCLC-BM, K^trans^, Non-Small Cell Lung Cancer Brain Metastasis; Volume Transfer Constant; K_ep_, Rate Constant; Ve, Vascular Extracellular Volume Fraction; Vp, Plasma Volume Fraction;iAUC_90_, Initial area under the curve at 90 seconds; CER, Contrast Enhancement Ratio; Max. Slope, Maximum Slope; Delay, Delay Time. Bold values indicate statistically significant results.

**Table 3:** Histopathological differential diagnosis of LUAD-BM and LUSC-BM.

Parameters	AUC	95% CI	*P*	Cutoff value	Sensitivity	Specificity
ADC(10^-6^mm²/s)	**0.534**	**0.445-0.623**	**0.4293**	**1437**	**30.30%**	**86.03%**
K^trans^(min^-1^/1000)	**0.998**	**0.995-1.000**	**<0.0001**	**157**	**98.48%**	**97.79%**
K_ep_(min^-1^/1000)	**0.962**	**0.936-0.987**	**<0.0001**	**727**	**93.94%**	**86.03%**
Ve(1/1000)	**0.848**	**0.795-0.901**	**<0.0001**	**223.6**	**80.30%**	**73.53%**
Vp(1/1000)	**0.976**	**0.957-0.994**	**<0.0001**	**34.90**	**93.94%**	**90.44%**
iAUC90(min·mmol/L/100)	**0.938**	**0.896-0.981**	**<0.0001**	**37.75**	**84.85%**	**96.32%**
CER(1/100)	**0.885**	**0.832-0.939**	**<0.0001**	**168.4**	**78.79%**	**88.24%**
Max.Slope (mmol·min^-^¹/100)	**0.953**	**0.915-0.991**	**<0.0001**	**124.1**	**87.88%**	**97.79%**
Delay(s)	**0.624**	**0.539-0.708**	**0.0044**	**6.15**	**45.45%**	**77.21%**

LUAD-BM, Lung Adenocarcinoma Brain Metastasis; LUSC-BM, Lung Squamous Cell Carcinoma Brain Metastasis; K^trans^, Volume Transfer Constant; K_ep_, Rate Constant; Ve,Vascular Extracellular Volume Fraction; Vp, Plasma Volume Fraction; iAUC_90_,Initial area under the curve at 90 seconds; CER, Contrast Enhancement Ratio; Max.Slope, Maximum Slope; Delay, Delay Time. Bold values indicate statistically significant results.

In differentiating SCLC-BM from NSCLC-BM, the ADC value demonstrated the highest efficacy (AUC = 0.891, p<0.0001). Using a cutoff value of 752.4 × 10^-6^ mm²/s, the sensitivity was 72.58% and specificity was 95.05%. A low ADC value (≤752.4 × 10^-6^ mm²/s) strongly indicated SCLC-BM. Among DCE-MRI parameters, Max. Slope exhibited good diagnostic capability (AUC = 0.813, p<0.0001), with 100% sensitivity and 72.28% specificity at a cutoff of 108.8 mmol·min^-^¹/100. iAUC_90_ also showed high diagnostic performance (AUC = 0.812), with 95.16% sensitivity and 70.30% specificity at a cutoff of 35.90 min·mmol/L/100. Both parameters achieved high sensitivity and specificity. CER and K^trans^ showed moderate diagnostic efficacy (AUC = 0.796 and 0.743, respectively). For CER, a cutoff of 171.7/100 yielded 98.39% sensitivity and 65.35% specificity. For K^trans^, a cutoff of 176.1 min^-^¹/1000 resulted in 93.55% sensitivity and 64.36% specificity. Ve and K_ep_ demonstrated limited diagnostic utility (AUC = 0.699 and 0.632, respectively), with K_ep_ showing high sensitivity (91.94%) but low specificity (37.13%).

In differentiating LUAD-BM from LUSC-BM, DCE-MRI parameters exhibited superior reliability. The K^trans^ parameter achieved near-perfect diagnostic performance (AUC = 0.998, p<0.0001), with 98.48% sensitivity and 97.79% specificity at a cutoff of 157 min^-^¹/1000, establishing it as the core diagnostic indicator. Vp and Max. Slope also demonstrated excellent efficacy (AUC = 0.976 and 0.953, respectively). For Vp, a cutoff of 34.90/1000 resulted in 93.94% sensitivity and 90.44% specificity. For Max. Slope, a cutoff of 124.1 mmol·min^-^¹/100 yielded 87.88% sensitivity and 97.79% specificity. K_ep_ and iAUC_90_ also performed notably well (AUC = 0.962 and 0.938, respectively). At cutoffs of 727 min^-^¹/1000 and 37.75 min·mmol/L/100, they achieved sensitivities of 93.94% and 84.85%, and specificities of 86.03% and 96.32%. Ve and CER parameters showed moderate efficacy (AUC = 0.848 and 0.885, respectively), with sensitivities of 80.30% and 78.79% and specificities of 73.53% and 88.24% at cutoffs of 223.6/1000 and 168.4/100. In contrast, the ADC value (AUC = 0.534, p=0.4293) lacked statistical significance and was ineffective for differentiating LUAD-BM from LUSC-BM.

In summary, the ADC value is highly effective for distinguishing SCLC-BM from NSCLC-BM, while DCE-MRI parameters such as K^trans^ and Vp are exceptionally reliable for differentiating LUAD-BM from LUSC-BM. These findings provide critical imaging-based evidence for noninvasive pathological classification in clinical practice.

### Quantifying the contributions of ADC values and DCE-MRI parameters to different histopathological LCBM subtypes using logistic regression

By constructing a multivariate logistic regression model adjusted for parameters including K^trans^, Ve, Vp, K_ep_, iAUC_90_, CER, Max. Slope, and Delay, the independent predictive effects of these indicators on different histopathological subtypes of LCBM exhibited significant heterogeneity, as detailed in **Table 4**.

**Table 4:** Logistic regression assessment of the contributions of ADC Values and DCE-MRI parameters to different histopathological subtypes of LCBM.

Parameters	LUAD-BM				LUSC-BM				SCLC-BM			
	β	OR	95%CI	*P*	β	OR	95%CI	*P*	β	OR	95%CI	*P*
ADC(10^-6^mm²/s)	**0.002**	**1.002**	**0.998~1.007**	**0.305**	**0.009**	**1.009**	**1.005~1.015**	**0.0001**	**-0.007**	**0.994**	**0.990~0.996**	**<0.0001**
K^trans^(min^-1^/1000)	**0.038**	**1.039**	**1.004~1.097**	**0.051**	**-0.105**	**0.901**	**0.837~0.953**	**0.001**	**-0.012**	**0.984**	**0.964~1.000**	**0.081**
K_ep_(min^-1^/1000)	**0.006**	**1.006**	**1.001~1.014**	**0.050**	**-0.002**	**0.998**	**0.991~1.003**	**0.532**	**0.004**	**1.004**	**1.001~1.008**	**0.015**
Ve(1/1000)	**0.003**	**1.003**	**0.964~1.039**	**0.851**	**0.009**	**1.009**	**0.989~1.029**	**0.348**	**0.010**	**1.010**	**0.998~1.024**	**0.116**
Vp(1/1000)	**0.270**	**1.310**	**1.121~1.669**	**0.005**	**-0.076**	**0.927**	**0.857~0.993**	**0.041**	**0.068**	**1.070**	**1.014~1.150**	**0.029**
iAUC90 (min·mmol/L/100)	**-0.110**	**0.896**	**0.582~1.399**	**0.594**	**0.275**	**1.317**	**1.033~1.743**	**0.036**	**-0.016**	**0.984**	**0.828~1.148**	**0.848**
CER(1/100)	**0.030**	**1.030**	**0.998~1.079**	**0.098**	**-0.056**	**0.945**	**0.914~0.971**	**0.0002**	**0.039**	**1.040**	**1.018~1.069**	**0.002**
Max.Slope (mmol·min^-^¹/100)	**0.079**	**1.082**	**0.951~1.258**	**0.232**	**0.059**	**1.061**	**1.009~1.129**	**0.034**	**-0.099**	**0.910**	**0.848~0.951**	**0.001**
Delay(s)	**1.453**	**4.278**	**1.078~31.20**	**0.078**	**0.362**	**1.436**	**0.847~2.644**	**0.203**	**-0.872**	**0.418**	**0.232~0.689**	**0.002**

DCE-MRI, Dynamic Contrast-Enhanced Magnetic Resonance Imaging; ADC, Apparent Diffusion Coefficient; LCBM, Lung Cancer Brain Metastasis; LUAD-BM, Lung Adenocarcinoma Brain Metastasis; LUSC-BM, Lung Squamous Cell Carcinoma Brain Metastasis; SCLC-BM, Small Cell Lung Cancer Brain Metastasis; K^trans^, Volume Transfer Constant; K_ep_, Rate Constant; Ve, Vascular Extracellular Volume Fraction; Vp, Plasma Volume Fraction; iAUC_90_,Initial area under the curve at 90 seconds; CER, Contrast Enhancement Ratio; Max.Slope, Maximum Slope; Delay,Delay Time. Bold values indicate statistically significant results.

For LUAD-BM, only Vp emerged as an independent positive predictive factor (β=0.270, OR = 1.310, 95% CI: 1.121–1.669, p=0.005). Other parameters, such as ADC value (β=0.002,p=0.305) and K^trans^ (β=0.038,p=0.051), showed a positive trend but failed to reach statistical significance after adjustment.

In LUSC-BM, K^trans^ was identified as an independent negative predictive factor (β=−0.105, OR = 0.901, 95% CI: 0.837–0.953, p=0.001). This, combined with the positive predictive effect of ADC value (β=0.009, OR = 1.009, 95% CI: 1.005–1.015, p=0.0001), suggests the characteristic features of squamous carcinoma: low vascular permeability and moderate cellular density. Furthermore, the independent contributions of CER (β=−0.056OR = 0.945, p<0.0002) and Max. Slope (β=0.059, OR = 1.061, p=0.034) indicated their potential to assist in evaluating vascular function in LUSC-BM.

For SCLC-BM, ADC value was an independent negative predictor (β=−0.007, OR = 0.993, 95% CI: 0.990–0.996, p<0.0001), highlighting high cellular density as a core feature. This necessitates caution regarding drug permeation limitations due to compressed extracellular space. Additionally, the independent positive predictions of K_ep_ (β=0.004, OR = 1.004, 95% CI: 1.001–1.008, p=0.015) and Ve (β=0.010, OR = 1.010, p=0.116), along with associations with enhancement curve parameters—Delay (β=−0.872, OR = 0.418, p=0.002), Max. Slope (β=−0.099, OR = 0.910, p=0.001), and CER (β=0.039, OR = 1.040, p=0.002).

### Impact of DCE-MRI parameters on ADC values in different pathological types of LCBM: a multivariate linear regression analysis

Multivariate linear regression analysis revealed distinct pathological subtype-specific influences of DCE-MRI parameters on ADC values in LCBM. as detailed in **Table 5**.

**Table 5:** Linear regression: pathology-specific effects of DCE-MRI parameters on ADC Values.

Rameters	LUAD-BM			LUSC-BM			SCLC-BM		
	*β*	*T*	*P*	*β*	*T*	*P*	*β*	*T*	*P*
K^trans^(min^-1^/1000)	-0.685	2.131	**0.035**	16.55	4.774	**<0.0001**	-2.685	1.669	0.101
K_ep_(min^-1^/1000)	0.417	5.976	**<0.0001**	-1.638	3.719	**0.0005**	0.361	1.478	0.145
Ve(1/1000)	0.311	1.603	0.112	-4.685	2.740	**0.0082**	1.448	1.535	0.131
Vp(1/1000)	2.294	1.299	0.196	-16.66	3.264	**0.0019**	-12.39	1.503	0.139
iAUC90(min·mmol/L/100)	4.823	1.468	0.145	-49.22	2.912	**0.0051**	24.81	1.118	0.269
CER(1/100)	-0.400	1.334	0.185	1.941	2.949	**0.0046**	1.620	1.280	0.206
Max.Slope (mmol·min^-^¹/100)	-0.471	0.413	0.680	8.903	1.700	0.0946	-7.290	1.960	0.055
Delay(s)	146.0	5.463	**<0.0001**	72.67	1.753	0.0850	-48.25	1.391	0.170

DCE-MRI, Dynamic Contrast-Enhanced Magnetic Resonance Imaging; ADC, Apparent Diffusion Coefficient; LUAD-BM, Lung Adenocarcinoma Brain Metastasis; LUSC-BM, Lung Squamous Cell Carcinoma Brain Metastasis; SCLC-BM, Small Cell Lung Cancer Brain Metastasis;K^trans^,Volume Transfer Constant; K_ep_, Rate Constant; Ve, Vascular Extracellular Volume Fraction; Vp, Plasma Volume Fraction; iAUC_90_,Initial area under the curve at 90 seconds; CER, Contrast Enhancement Ratio; Max.Slope, Maximum Slope; Delay, Delay Time. Bold values indicate statistically significant results.

For LUAD-BM, ADC values exhibited significant positive regulation by the Kep (β=0.417, p < 0.0001) and Delay(β=146.0,p<0.0001), while showing weak negative association with K^trans^ (β=−0.685, p=0.035). Parameters including Ve(β=0.311), Vp (β=2.294), iAUC_90_ (β=4.823), CER (β=−0.400), and Max.Slope (β=−0.471) demonstrated no statistical significance (all p > 0.05).

In LUSC-BM, ADC values were positively driven by K^trans^ (β=16.55, p<0.0001) and CER (β=1.941,p=0.005),but negatively associated with Ve(β=−4.685,p=0.008),Vp(β=−16.66,p=0.002),iAUC_90_(β=−49.22,p=0.005),and K_ep_ (β=−1.638, p=0.001), with Max.Slope approaching significance (β=8.903, p=0.095).

SCLC-BM displayed only marginal negative correlation between Max.Slope and ADC (β=−7.290, = 0.055), while other parameters (K^trans^, K_ep_, Ve, Vp, iAUC_90_, CER, Delay) showed no significant associations (all p>0.05).

### Impact of DCE-MRI parameters and ADC values on BBB leakage in different pathological types of LCBM: a multivariate linear regression analysis

Multivariate linear regression analysis demonstrated significant pathological subtype-specific effects of DCE-MRI parameters and ADC values on BBB permeability (K^trans^)in LCBM.as detailed in **Table 6**.

**Table 6:** Linear regression- effects of DCE-MRI and ADC parameters on blood-brain barrier permeability in different histopathological types of LCBM.

Parameters	LUAD-BM			LUSC-BM			SCLC-BM		
	*β*	*T*	*P*	*β*	*T*	*P*	*β*	*T*	*P*
ADC(10^-6^mm²/s)	-0.050	2.131	0.035	0.012	3.719	0.001	-0.019	1.669	0.101
K_ep_(min^-1^/1000)	0.105	5.436	<0.0001	0.114	14.54	<0.0001	0.1203	9.611	<0.0001
Ve(1/1000)	0.390	9.657	<0.0001	0.282	14.99	<0.0001	0.1254	1.599	0.116
Vp(1/1000)	2.182	4.938	<0.0001	0.614	4.314	<0.0001	-3.323	6.246	<0.0001
iAUC90(min·mmol/L/100)	1.151	1.290	0.200	1.528	3.523	0.001	7.753	5.050	<0.0001
CER(1/100)	0.227	2.852	0.005	0.041	1.315	0.194	0.352	3.694	0.001
Max.Slope (mmol·min^-^¹/100)	0.353	1.146	0.254	-0.317	2.388	0.020	0.093	0.291	0.773
Delay(s)	-15.11	1.899	0.060	-1.494	1.686	0.097	6.775	2.430	0.019

K^trans^, defined as the Volume Transfer Constant (from blood plasma to extravascular extracellular space), is used to quantify blood-brain barrier (BBB) permeability.

DCE-MRI, Dynamic Contrast-Enhanced Magnetic Resonance Imaging; ADC, Apparent Diffusion Coefficient; BBB, Blood-Brain Barrier; LCBM, Lung Cancer Brain Metastasis; LUAD-BM, Lung Adenocarcinoma Brain Metastasis; LUSC-BM, Lung Squamous Cell Carcinoma Brain Metastasis; SCLC-BM, Small Cell Lung Cancer Brain Metastasis; K_ep_, Rate Constant; Ve, Vascular Extracellular Volume Fraction; Vp, Plasma Volume Fraction; iAUC_90_,Initial area under the curve at 90 seconds; CER, Contrast Enhancement Ratio; Max.Slope, Maximum Slope; Delay, Delay Time.

In LUAD-BM, K_ep_(β=0.105,p<0.0001),Ve(β=0.390,p<0.0001),and Vp(β=2.182,p<0.0001) exhibited significant positive associations with K^trans^, while reduced ADC values (β=−0.050,p=0.035) and elevated CER(β=0.227,p=0.005) increased BBB permeability. Parameters including iAUC90(β=1.151,p=0.200), Max.Slope(β=0.353,p=0.254),and Delay(β=−15.11,0p=0.060) showed no statistical significance.

For LUSC-BM, K^trans^ was positively driven by K_ep_(β=0.114p<0.0001), Ve (β=0.282,p<0.0001), and Vp(β=0.614, p<0.0001). Increased ADC values (β=0.012,p=0.001) and iAUC_90_ (β=1.528,p=0.001) enhanced BBB permeability, whereas Max.Slope reduction correlated with decreased K^trans^ (β=−0.317,p=0.020). CER (β=0.041,p=0.194) and Delay (β=−1.494,p=0.097) lacked significant contributions.

In SCLC-BM, K^trans^ was prominently enhanced by K_ep_ (β=0.1203,p<0.0001) and iAUC_90_ (β=7.753,p<0.0001), with CER further elevating permeability (β=0.352,p=0.001). Notably, Vp reduction (β=−3.323,p<0.0001) and prolonged Delay (β=6.775,p=0.019) revealed unique regulatory mechanisms. ADC (β=−0.019,p=0.101), Ve(β=0.1254,p=0.116), and Max.Slope (β=0.093,p=0.773) showed no significant effects. Collectively, BBB permeability regulation exhibited pathology-dependent patterns: LUAD-BM was dominated by microvascular function (Vp) and extracellular space (Ve); LUSC-BM relied on vascular transport kinetics (K_ep_) and enhancement kinetics (iAUC_90_); SCLC-BM demonstrated antagonistic effects between abnormal contrast agent kinetics (Kep/iAUC_90_) and microvascular density (Vp).

## Discussion

The pathophysiological heterogeneity of LCBM, encompassing factors such as BBB disruption, vascularization patterns, and extracellular matrix remodeling, constitutes a critical constraint on therapeutic efficacy (17). Traditional pathological biopsies, limited by their invasiveness, sampling bias, and postoperative diagnostic delays, fail to comprehensively and dynamically assess the tumor microenvironment. This study innovatively integrates vascular functional parameters from DCE-MRI with DWI to establish a “vascular-cellular microenvironment visualization model” for LCBM. Through multiparametric fusion analysis, we systematically characterized the microenvironmental profiles of distinct histopathological LCBM subtypes: LUAD-BM exhibited high microvascular density and vascular permeability; LUSC-BM was defined by low vascular permeability and moderate cellular density; SCLC-BM demonstrated high cellular density and anomalous contrast agent transport kinetics. These findings not only validate the capability of multimodal functional imaging parameters to characterize LCBM microenvironmental heterogeneity but also provide novel insights into tumor vascularization, cellular density, and BBB regulation. Specifically, the quantification of independent parameter contributions (e.g., the independent positive predictive role of Vp for LUAD-BM in logistic regression, β=0.270, p=0.005) and interactive mechanisms (e.g., the synergistic regulation of LUSC-BM ADC values by K^trans^-driven enhancement and Ve mediated suppression in linear regression) establishes an imaging foundation for personalized therapeutic strategies targeting the tumor microenvironment.

The observed differences in imaging parameters among LCBM fundamentally reflect the heterogeneity of their vascular-cellular microenvironment. Through multiparametric fusion analysis of DCE-MRI and DWI, this study systematically delineates the imaging characteristics of the vascular-cellular microenvironment in LCBM. Key findings include: The ADC value demonstrated exceptional efficacy in discriminating SCLC-BM from NSCLC-BM (AUC = 0.891), achieving 95.05% specificity at a cutoff of 752.4×10^-6^ mm²/s, thereby providing a reliable indicator for identifying SCLC-BM with high cellular density. For distinguishing LUAD-BM from LUSC-BM, K^trans^ exhibited near-perfect diagnostic performance (AUC = 0.998), with sensitivity and specificity exceeding 97% at a cutoff of 157 min^-^¹/1000, confirming vascular permeability as a core stratification indicator. Mechanistically, logistic regression quantified Vp as an independent positive predictor for LUAD-BM (β=0.270, OR = 1.310, 95% CI: 1.121–1.669, p=0.005), validating its biological essence of “microvascular richness.” Conversely, K^trans^ served as an independent negative predictor for LUSC-BM (β=-0.105, OR = 0.901, 95% CI: 0.837–0.953, p=0.001), Combined with the positive association of ADC value (β=0.009, OR = 1.009, 95% CI: 1.005–1.015, p<0.0001), this indicates a microenvironment characterized by low vascular permeability and moderate cellular density. The independent contributions of CER (β=-0.056, p<0.0002) and Max.Slope (β=0.059, p=0.034) further corroborate the pathologically restricted vascular function in LUSC-BM. For SCLC-BM, ADC value emerged as an independent negative predictor (β=-0.007, OR = 0.993, 95% CI: 0.990–0.996, p<0.0001), while positive trends were observed for K_ep_ (β=0.004, OR = 1.004, 95% CI: 1.001–1.008, p=0.015) and Ve (β=0.010, OR = 1.010, p=0.116), quantitatively capturing the biological drivers of its “high cellular density and rapid contrast agent transport” phenotype and highlighting high cellular density as a defining feature. Multivariate linear regression further elucidated parameter interactions: LUAD-BM ADC values were primarily positively regulated by K_ep_ (β=0.417, p<0.0001) and Delay (β=146.0, p<0.0001), suggesting that vascular transport kinetics and contrast arrival time indirectly modulate cellular density via extracellular space pressure – consistent with LUAD-BM’s abundant microvasculature and large extracellular volume (median Ve=294.3). LUSC-BM ADC values showed positive association with K^trans^ (β=16.55, p<0.0001) but were negatively constrained by Ve (β=-4.685, p=0.008) and Vp (β=-16.66, p=0.002), reflecting fibrotic matrix-mediated restriction of microvascular formation (low Vp) and reduced extracellular space (low Ve), leading to elevated extracellular pressure and ADC limitation, thereby forming a “low vascular permeability-high matrix density” regulatory interplay. SCLC-BM exhibited minimal association between ADC and DCE-MRI parameters, with only marginal negative correlation to Max.Slope (β=-7.290, p=0.055), confirming its proliferation-dominant pathology where cellular density is primarily driven by intrinsic proliferative activity rather than vascular function, aligning with extracellular space compression (low ADC) and passive microvascular damage (high Kep) resulting from high proliferative activity. Analysis of BBB permeability (K^trans^) revealed distinct drivers: LUAD-BM showed strong positive associations with K_ep_ (β=0.105, p<0.0001), Ve (β=0.390, p<0.0001), and Vp (β=2.182, p<0.0001), and negative association with ADC (β=-0.050), indicating that rich microvascular content (high Vp), large extracellular volume (high Ve), and rapid transport kinetics (high K_ep_) are core drivers of its high BBB leakage. In LUSC-BM, BBB leakage was primarily maintained by limited microvascular function (low Vp), evidenced by positive drives from K_ep_ (β=0.114, p<0.0001), Vp (β=0.614, p<0.0001), and ADC (β=0.010). SCLC-BM demonstrated prominent positive contributions from K_ep_ (β=0.1203, p<0.0001) and iAUC_90_ (β=7.753, p<0.0001), coupled with strong negative association with Vp (β=-3.323, p<0.0001), suggesting its BBB leakage is governed by abnormal contrast transport patterns (high K_ep_, high iAUC_90_) rather than microvascular content (low Vp). Collectively, these differences demonstrate that distinct pathological subtypes of LCBM achieve functional regulation through specific parameter combinations (e.g., “high Vp + high Ve + high K_ep_” in LUAD-BM; “high Kep + high iAUC_90_ + low Vp” in SCLC-BM), providing quantitative evidence for deciphering the pathophysiological heterogeneity of LCBM. This study not only validates the hypothesis that multiparametric functional imaging reflects LCBM microenvironment heterogeneity but also, by quantifying independent parameter contributions and interactive relationships, offers novel insights into the biological mechanisms underlying tumor angiogenesis, cellular density, and dynamic BBB alterations, thereby establishing an imaging foundation for developing individualized therapies targeting the microenvironment.

This study confirms the robust diagnostic efficacy of ADC values in differentiating SCLC-BM from NSCLC-BM, aligning with prior reports (18, 19) that ADC effectively distinguishes high-cellular-density SCLC from other subtypes. Simultaneously, K^trans^ demonstrated near-perfect discriminative capability for LUAD-BM versus LUSC-BM, corroborating Abreu et al. (7) who identified K^trans^ as a pivotal biomarker of vascular permeability differences critical for LUAD/LUSC differentiation. Regarding the vascular-cellular microenvironment across pathological subtypes, LUAD-BM exhibited elevated vascular permeability, consistent with findings by Gozde Uzunalli et al. (4) and Du et al. (20). Du et al. attributed this to VEGF-driven angiogenesis, while Gozde Uzunalli et al. implicated EGFR mutation-mediated HIF-1α upregulation in immature vascularization. Our multiparameter logistic regression further quantified LUAD-BM’s “microvascular enrichment,” evidenced by Vp’s independent positive prediction, reinforcing Wei et al.’s (12) conclusion on microvascular density as a hallmark of adenocarcinoma. Crucially, unlike previous studies, we identified that LUAD-BM’s K^trans^ is co-driven by K_ep_, Ve, and Vp while ADC negatively modulated Ktrans, suggesting reduced ADC (high cellular density) may enhance BBB leakage—a novel insight into cellular-vascular feedback.LUSC-BM displayed low K^trans^, echoing Lang et al. (21) who attributed reduced permeability in spinal metastases to fibrotic matrix-impaired angiogenesis. This aligns with our observation of moderate ADC values in LUSC-BM. Mechanistically, Gozde Uzunalli et al. (4) linked BTB evolution to basement membrane degradation and pericyte dysfunction, complementing our findings: Ve negatively suppressed ADC in LUSC-BM, while SCLC-BM showed aberrantly elevated K_ep_, collectively highlighting microenvironmental specificity.For SCLC-BM, Zhang Bin et al. (22) reported significantly lower ADC than NSCLC-BM, with low ADC percentiles inversely correlating with Ki-67 (r=–0.521), strongly supporting our data: ADC independently predicted SCLC-BM negatively, while multivariate regression revealed minimal DCE-MRI influence. This limitation likely stems from extracellular space compression due to high proliferative activity or intrinsic proliferation-driven cellular density decoupled from vascular function—consistent with Huang et al. (23) who noted Ve-Ki-67 inverse correlation and Makoto et al. (18) reporting high cellular density in SCLC-BM. However, diverging from Makoto et al.’s observation of low vascular density (CD31+), our data showed SCLC-BM’s Vp exceeded LUSC-BM’s, potentially reflecting sampling bias in prior studies with imbalanced subtype representation.Additionally, while Ye et al. (24) reported deficient BBB/BTB efflux pumps (e.g., P-gp) in SCLC-BM, our observed high K_ep_ (indicating rapid contrast reflux) appears contradictory. Critically, these mechanisms differ fundamentally: efflux pumps mediate active drug transport, whereas contrast reflux reflects passive extravasation governed by vascular leakage and extracellular space heterogeneity. This distinction necessitates future investigation into the interplay between vascular permeability parameters (e.g., K_ep_) and active transport functionality in SCLC-BM.

The findings of this study hold direct clinical implications for the management of LCBM. In diagnostics, the high specificity of the ADC value in distinguishing SCLC-BM can circumvent the risks of invasive biopsy and prevent treatment delays. The near-perfect discriminative power of K^trans^ for differentiating LUAD-BM from LUSC-BM provides a non-invasive preoperative subtyping tool, particularly critical for inoperable patients, thereby optimizing clinical decision-making. For therapeutic strategies, the independent positive prediction of Vp for LUAD-BMsuggests prioritizing anti-angiogenic agents in lesions with rich microvasculature. Conversely, the low ADC value and high K_ep_ in SCLC-BM indicate compressed extracellular spaces that may limit drug penetration, necessitating combination with small-molecule targeted therapies or adjusted chemotherapy dosing. The low K^trans^ in LUSC-BM supports localized radiotherapy over systemic anti-angiogenic therapy. Diagnostic pitfalls, such as overreliance on single parameters (e.g., ADC’s inability to distinguish adenocarcinoma from squamous carcinoma) or overlooking microenvironmental interactions (e.g., opposing effects of K^trans^ and Ve on ADC in LUSC-BM), must be addressed to avoid misclassification or suboptimal treatment responses.At the pathophysiological level, the results validate established theories: LUAD-BM’s high K^trans^ aligns with VEGF-mediated angiogenesis (20), while ADC regulation by K_ep_ and Delay reflects indirect effects of vascular transport kinetics on cellular density via extracellular pressure. SCLC-BM’s ADC independence from DCE-MRI parameters confirms its proliferation-driven (non-angiogenic) pathology (24). The fibrotic matrix in LUSC-BM, restricting angiogenesis (low Vp, low Ve), aligns with the “stroma-vascular antagonism” model proposed by Chen et al. (13).

This study advances beyond prior descriptive approaches through a multiparametric fusion model with four key innovations:1)Integration of DCE-MRI vascular parameters and DWI structural metrics to construct a “vascular-cellular microenvironment visualization model,” systematically characterizing LUAD-BM (“high microvascular density + large extracellular space”), SCLC-BM (“high cellular density + aberrant vascular function”), and LUSC-BM (“low vascular permeability”) heterogeneity.2)Quantification of independent parameter contributions via logistic regression, identifying Vp as the sole independent positive predictor for LUAD-BM (β=0.270) and ADC as an independent negative predictor for SCLC-BM, transforming correlation into mechanistic insight.3)Elucidation of parameter interactions through multivariate linear regression, revealing LUAD-BM’s ADC dominance by K_ep_ and Delay, and LUSC-BM’s ADC dual regulation by K^trans^(positive) and Ve (negative).4)Incorporation of key thresholds (e.g., SCLC-BM ADC: 752.4 × 10^-6^ mm²/s; LUAD-BM K^trans^: 176.1 min^-^¹/1000) into clinical scoring systems (e.g., DS-GPA), enabling personalized strategies such as “anti-angiogenic therapy for high-Vp LUAD-BM” or “combination targeted therapy for low-ADC SCLC-BM,” thereby establishing an “imaging-pathology-clinical” framework that enhances translational impact.Collectively, this work not only validates multiparametric MRI for characterizing LCBM microenvironments but also provides novel theoretical and technical pathways for fundamental research and precision therapeutics through multimodal integration, parameter weighting, interaction analysis, and clinical application frameworks.

This study acknowledges several limitations: the relatively small sample size and single-center design necessitate caution when generalizing the results; lesions smaller than 0.5 cm were excluded based on inclusion criteria; manual ROI delineation may introduce observer bias despite achieving inter-observer ICC values ≥0.8; the absence of post-treatment follow-up data precludes validation of the parameters’ predictive value for therapeutic response; and potential confounding factors—including enhancement patterns, perilesional edema, spatial distribution of metastases, and genetic mutation status—were not systematically controlled. Furthermore, molecular biomarkers from both primary and metastatic sites remain unanalyzed, highlighting the need for future exploration of imaging-molecular correlations. Subsequent research should validate the model’s generalizability through multi-center studies with larger cohorts, integrate PET/CT or MR spectroscopy to supplement metabolic information, conduct longitudinal investigations to correlate parameter dynamics with clinical outcomes, and leverage AI-driven techniques to extract more sensitive microstructural features, thereby enhancing diagnostic efficacy.

## Conclusions

In summary, this study systematically revealed the vascular-cellular microenvironmental heterogeneity of LCBM through multiparametric fusion analysis of DCE-MRI and DWI. Key findings elucidated distinct pathophysiological characteristics: cellular density in SCLC-BM is predominantly driven by autonomous proliferation, LUAD-BM exhibits hyperactive vascular function, and LUSC-BM demonstrates restricted angiogenesis. These results hold significant implications across clinical and basic scientific domains. Clinically, they establish a reliable noninvasive imaging tool for pathological subtyping of LCBM, enabling optimized therapeutic decisions—such as prioritizing anti-angiogenic agents (e.g., bevacizumab) for LUAD-BM, selecting stereotactic radiotherapy for LUSC-BM, and identifying high-risk SCLC-BM cases via low ADC values. From a fundamental research perspective, this work deepens the mechanistic understanding of dynamic vascular-cellular microenvironmental regulation in LCBM, validates the theoretical hypothesis that multimodal functional imaging parameters can precisely characterize microenvironmental heterogeneity, and provides an imaging-based rationale for developing therapeutic strategies targeting critical pathological components such as the BBB/BTB, and extracellular matrix.

## Data Availability

The original contributions presented in the study are included in the article/supplementary material. Further inquiries can be directed to the corresponding authors.
